# Task instructions modulate the attentional mode affecting the auditory MMN and the semantic N400

**DOI:** 10.3389/fnhum.2014.00654

**Published:** 2014-08-27

**Authors:** Helena Erlbeck, Andrea Kübler, Boris Kotchoubey, Sandra Veser

**Affiliations:** ^1^Department of Psychology I, University of WürzburgWürzburg, Germany; ^2^Institute of Medical Psychology and Behavioral Neurobiology, University of TübingenTübingen, Germany

**Keywords:** ERP, attention, instruction, priming, N400, MMN

## Abstract

Event-related potentials (ERPs) have been proven to be a useful tool to complement clinical assessment and to detect residual cognitive functions in patients with disorders of consciousness. These ERPs are often recorded using passive or unspecific instructions. Patient data obtained this way are then compared to data from healthy participants, which are usually recorded using active instructions. The present study investigates the effect of attentive modulations and particularly the effect of active vs. passive instruction on the ERP components mismatch negativity (MMN) and N400. A sample of 18 healthy participants listened to three auditory paradigms: an oddball, a word priming, and a sentence paradigm. Each paradigm was presented three times with different instructions: ignoring auditory stimuli, passive listening, and focused attention on the auditory stimuli. After each task, the participants indicated their subjective effort. The N400 decreased from the focused task to the passive task, and was extinct in the ignore task. The MMN exhibited higher amplitudes in the focused and passive task compared to the ignore task. The data indicate an effect of attention on the supratemporal component of the MMN. Subjective effort was equally high in the passive and focused tasks but reduced in the ignore task. We conclude that passive listening during EEG recording is stressful and attenuates ERPs, which renders the interpretation of the results obtained in such conditions difficult.

## Introduction

The ability to attend to and focus on certain events in the environment forms the basis of all cognitive functions and mental processes. Thus, the concept of attention has been of great interest in investigating not only healthy individuals but also patients with cognitive impairment. In this regard, event-related potentials (ERPs) have been proven to be a successful tool in the study of attentional processes in general and as a complement to clinical assessment for detecting cognitive functions associated with attention (Kotchoubey et al., 2002, 2005). The allocation of attention to the relevant or concurrent stimuli, which can be easily modulated by a specific instruction, can substantially affect the amplitude, latency, and even the mere presence or absence of ERPs. However, at least in recordings with patients with severe disorders of consciousness (DoC), this aspect has rarely been considered and the instructions given are often passive or unspecific. However, there have already been specific calls for the use of active instructions (Kübler and Kotchoubey, 2007; Kotchoubey and Lotze, 2013). Severe DoC includes states of inhibited consciousness and self-awareness such as coma, unresponsive wakefulness syndrome (UWS), and minimally conscious state (MCS). However, locked-in syndrome (LIS), which is characterized by preserved awareness but the inability to communicate by movement, is not a DoC but can be confused with DoC when assessed using behavioral tasks. Patients in coma, with UWS, MCS, or LIS are largely or completely unresponsive in their motor reactions but may have different states of awareness of themselves and their environment. Since ERPs provide information that allows us to assess cognitive functions in DoC patients, the extent to which attention allocation is modulated in active vs. passive tasks is particularly relevant. In terms of clinical assessment, ERPs can help to extract additional information about the current cognitive state of a DoC patient but cannot be included in the diagnostic processes because of their lack of standardization. In fact, only positive ERP results can be interpreted. Negative results, however, can be caused by various factors, such as varying states of arousal, focus of attention, language understanding, etc., and all these factors affect attention allocation to the stimuli of interest. This study examines the effect that attentional modulation has on two different ERP components, i.e., on mismatch negativity (MMN) and the semantic N400, in healthy participants and its relevance for the measurement and assessment of patient data.

The following section describes the theoretical background of MMN and N400 and reviews the literature about attentional variation of these ERP components. Further, the application of MMN and N400 paradigms is related to patient settings and finally, our hypotheses are defined.

The MMN belongs to a group of ERPs referred to as N200, which was first recorded by Sutton et al. (1965). The N200 can be further subdivided into the N2a or MMN, N2b, and N2c subcomponents, depending on the stimuli used, scalp distribution, and allocation of attention (Pritchard et al., 1991). An MMN is typically elicited in an oddball paradigm comprising one stimulus which occurs frequently (standard), and one that differs from this standard and occurs rarely and unpredictably (deviant). In the auditory domain, an MMN appears in response to deviants that vary in one or more stimulus features such as frequency, intensity, duration, or location (Näätänen et al., 2004). According to the memory-mismatch or trace-mismatch hypothesis, an MMN is elicited when an incoming stimulus differs from the memory representation formed by the preceding stimulus sequence (Näätänen, 1990). This concept was later challenged by the regulation violation hypothesis (Winkler, 2007), which postulates that the retention of auditory stimulation in memory represents regularity. However, the trace-mismatch and regulation violation hypotheses are not mutually exclusive (Kimura et al., 2010).

The MMN peaks between 150 and 250 ms after deviant onset and is obtained by subtracting the ERP response elicited by the standards from that elicited by the deviants. Furthermore, MMN amplitudes typically reverse polarity at the mastoid electrodes and this reversal can be used to differentiate the MMN from other potentials like N2b (Näätänen et al., 2007). According to the two-component model (Näätänen and Michie, 1979; Näätänen et al., 2007), one right-hemispheric frontal and one supratemporal component contribute to the MNN. It is assumed that amplitudes recorded at the mastoids represent an estimate of the mere supratemporal component without an overlap of N2b.

The MMN is often regarded as a response generated by an automatic change detection mechanism that occurs without conscious perception and independently of the attention of the listener (Näätänen, 1990; Muller-Gass et al., 2005; Folstein and Van Petten, 2007). However, this contradicts findings that under some conditions, the MMN amplitude can be either enhanced by directing attention toward a discrimination task (Woods et al., 1992; Oades and Dittmann-Balcar, 1995), or attenuated by strongly focusing attention toward some other (irrelevant) stimuli (Woldorff et al., 1991, 1998). To resolve this discrepancy, Sussman proposed that two steps are necessary to elicit an MMN (Sussman, 2007): standard formation and deviance detection. She argues that only the first process, the formation of a standard memory trace, is directly affected by attention. An acoustic stimulus becomes a standard through repetition and is then maintained in the auditory memory. This process establishes the basis of the second process, detection of the deviant, which fully relies on the representations formed by the standard and is fairly indifferent to attention. Thus, the MMN is not a pre-attentive process but a part of a larger system of auditory scene analysis consisting of interacting sub-processes, which can be modulated by attention (Sussman, 2007).

The N400 occurs as slow monophasic negativity between 200 and 600 ms and is mainly regarded as a specific response to violations of semantic expectations (Kutas and Hillyard, 1980). It occurs in response to congruent vs. incongruent sentence endings (Kutas and Hillyard, 1984; Kutas, 1987), and related vs. unrelated word pairs (Bentin et al., 1985; Hagoort et al., 1996), as well as to line drawings completing a sentence (Ganis et al., 1996), incongruent endings of picture stories (West and Holcomb, 2002), and inappropriate objects in video films (Sitnikova et al., 2003). However, the N400 has also been observed in response to pseudowords with no relation to real words (Deacon et al., 2004), which implies that specific semantic meaning is not a necessary condition for its elicitation. Since the N400 does not need to be negative in absolute terms, the amplitude is calculated as the difference between responses to congruent and incongruent stimuli (Kutas and Federmeier, 2011).

The N400 could represent an automatic or controlled mechanism of semantic processing. Attenuation of the N400 in response to unattended targets varies considerably (McCarthy and Nobre, 1993; Kutas and Federmeier, 2011). Masking of prime words, which makes them less perceptible and reportable, did attenuate the N400 in a word priming paradigm; however, masking did not eliminate it (Holcomb and Grainger, 2009). In contrast, just a moderate masking completely suppressed the N400 in a sentence paradigm (Daltrozzo et al., 2012; for a review, see Kutas and Federmeier, 2011). It seems likely that the N400 comprises characteristics of both, automatic and controlled processing. Since the role of attention in eliciting the N400 is not yet completely understood, it is particularly important to be able to estimate attentional effects on the N400 component, especially if the presence or absence of this kind of ERP component is to be used to test cognitive functioning of DoC patients.

Attention processes measured by ERPs take on special practical importance when it comes to the diagnosis and treatment of patients with severe cognitive impairment such as DoC caused by head injury, stroke, or anoxia (Ilvonen, 2003; Kotchoubey et al., 2005; Schnakers et al., 2008). Studies indicated that the presence of an MMN is a strong predictor for awakening from coma and UWS (Fischer et al., 2004; Wijnen et al., 2007; for a review see Daltrozzo et al., 2007). Similarly, N400 effects indicated preserved semantic processing in some DoC patients (Schoenle and Witzke, 2004; Kotchoubey et al., 2005). In addition, the N400 has been shown to be a predictor of recovery in UWS and MCS patients (Faran et al., 2006; Steppacher et al., 2013) and a correlate of quality of life and thus, successful coping in patients with amyotrophic lateral sclerosis (Real et al., 2014).

In the present study, we expected that different attentional instructions affect the elicitation of the N400 and MMN. Although we can rarely know the current state of consciousness in DoC patients, measurements are often carried out assuming that there is at least residual awareness. On the basis of this assumption, the importance of appropriate instructions to record reliable ERPs is evident. Since there are significant differences in the instructions for patients, we aimed at clarifying how N400 and MMN, two ERPs with great potential to be used in patient assessment, are influenced by attentional modulations in healthy participants.

In the present study, we investigated the effect of attentional modulation on ERPs obtained in three different paradigms (one oddball, two semantic). This investigation is unique in three aspects. Firstly, we included the discrete effect of a behaviorally passive attentional instruction to modulate attention. This instruction was covert and unspecific such that no overt responses to the auditory stimuli were required. We compared three different attentional situations: ignoring auditory stimuli, passive attention, and focused attention. Secondly, we investigated both, a simple oddball paradigm, and complex semantic paradigms recorded in identical tasks. Thirdly, we complemented ERP findings with subjective ratings of the effort experienced after each task and paradigm.

We expected the ERP components and subjective effort to vary according to the manipulation of attention. The focused task required fast and correct reactions to the auditory stimuli and was thus expected to be the most strenuous. The ignore task only required few key presses in reaction to the presentation of predefined scenes in a movie of emotionally neutral content which was expected to be the least strenuous. The passive task, however, did not require any overt response; therefore the participant was unable to engage in a specific activity. This state may cause boredom accompanied by feelings of negative affect and mental effort (Eastwood et al., 2012). Thus, we expected the average subjective effort to be highest in the task with focused attention (focused task), lower in the passive attention task (passive task), and lowest in the task in which attention was drawn away (ignore task), irrespective of the paradigm.

On the physiological level, we expected an N400 effect in both semantic paradigms. This effect was assumed to be greatest in the focused task, diminished in the passive task, and most strongly attenuated in the ignore task referring to previously found attention effects in the N400 component (McCarthy and Nobre, 1993; Chwilla et al., 1995). In the oddball paradigm, we assumed a similar modulation of the MMN effect. We expected this variation of the MMN because of the assumed effect of the attentional instruction on the standard formation process as postulated by Sussman (2007).

## Methods

### Participants

We recorded EEG in 18 healthy adults (seven males, two left-handed) at the University of Würzburg. The participants were between 25 and 49 years old (mean = 33.39, *SD* = 7.55 years) and received an expense allowance of EUR 9 per hour. All participants had normal hearing and were not in treatment for any psychiatric or neurological disorders at the time of the study. All participants gave their written consent after they were informed about the nature of the study. The study was conducted in accordance with the Declaration of Helsinki and was approved by the Ethical Review Board of the Medical Faculty, University of Würzburg.

### Experimental procedure and stimuli

The present study comprised three tasks (ignore, passive, focused) and three paradigms (oddball, word pairs, sentences). In this respect, the term “task” is defined as the instruction given to the participants, whereas “paradigm” describes the auditory stimulation being presented. All paradigms were combined with all tasks, resulting in a 3 × 3 experimental design.

The experimental procedure consisted of three auditory paradigms: (a) an oddball paradigm, (b) a word priming paradigm, and (c) a sentence paradigm. Each paradigm was presented three times with three different attention tasks. The tasks were presented in a pseudorandom order such that the passive task always preceded the focused task. The paradigms were also presented pseudorandomly within the tasks such that consecutive paradigms were never identical. In the ignore task, the presentation of auditory stimuli was accompanied by a documentary silent movie (Vertov, 2006) which did not require any language to follow the content. The participants' task was to press a key when a certain scene appeared. The movie was cut into three parts containing between 20 and 24 occurrences of the relevant scene (2 s long) at intervals of 15–45 s. These sections were presented in random order so that each paradigm was accompanied by a different part of the movie. The instructions were given in German and contained the following information: “In the following experiment, you will hear tones/semantically right or wrong sentences/related and unrelated word pairs and see a silent movie. Your task is to watch out for a specific scene in the movie and press the “M” key as soon as the scene appears. Please look out for the following scene. [scene is shown]. Are you ready? The experiment starts in a few seconds.” In the passive task, the participants were instructed to just listen to auditory stimuli. The exact instruction was: “In the following experiment, you will hear tones/semantically right or wrong sentences/related and unrelated word pairs. Please just listen and watch the fixation cross in the center of the screen. Are you ready? The experiment starts in a few seconds.” In the focused task, the participants were required to indicate either the odd tone or semantically congruent and incongruent stimuli by pressing a key (oddball) or two different keys (semantic paradigms). The exact instruction was: “In the following experiment, you will hear tones/semantically right or wrong sentences/related and unrelated word pairs. Please listen carefully and press the “M” key as soon as you detect a deviant tone/press the “M” key for related word pairs/semantically correct sentences and the “B” key for unrelated word pairs/semantically incorrect sentences. Are you ready? The experiment starts in a few seconds.”

The oddball paradigm comprised 1000 three-component harmonic sounds of 440+880+1760 Hz, with 900 standard stimuli with a duration of 50 ms and 100 deviant stimuli with a duration of 20 ms. The inter-stimulus interval between the onset of two successive tones was 350 ms. The first five tones were always standards and a deviant was always followed by a standard. The word priming paradigm comprised 100 semantically related (e.g., mountain-valley) and 100 semantically unrelated word pairs (e.g., place-bravery). The inter-stimulus interval was 500 ms within and 1500 ms between the word pairs. The word pairs were defined in a pre-experiment in which 45 participants rated the relation of various word pairs. Only related word pairs with a prime strength above 90% and unrelated word pairs with a prime strength below 10% were selected. The same words were used for the related and unrelated condition, thus each word was presented twice. The sentence paradigm comprised 200 short sentences of which 100 ended with an incorrect word (e.g., “The eel is a bird.”) and 100 with a correct word (e.g., “The eel is a fish.”). The inter-stimulus interval between the sentences was 1500 ms. The sentences were selected in the same pre-experiment as the word pairs. The participants rated the sentences as correct or incorrect and only sentences which were rated with a certainty above 90% were included. All correct end words also appeared as incorrect end words. All stimuli were spoken by a young female German native speaker with a clear voice free of any dialect inflection. All sounds had a sampling rate of 44.1 Hz, a resolution of 32 bits and were presented at a sound level of 65 dB(A).

All auditory stimuli were presented via pneumatic transducer in-ear headphones (3M™ E-A-RTONE™ Insert Earphone 3A ABR, 50 ohm) equipped with foam eartips (Etymotic research, inc., eartips for ER-3 and ER-5; Killion, 1984).

To evaluate the perceived effort, the participants indicated their subjective effort after each paradigm on a scale from 0 to 220 with seven labels ranging from “rarely strenuous” at 20 to “extraordinarily strenuous” at about 205 (Eilers et al., 1986). The participants could indicate their experienced effort on any position of the scale.

All the paradigms and tasks were presented within a single session. The absolute recording time was approximately 90 min; the whole experiment took between 2 and 3 h depending on breaks, further explanations, etc.

### Material and data acquisition

We recorded EEG according to the international 10–20 system with a BrainAmp ActiCap system (Brain Products, Gilching, Germany) using 32 Ag/AgCl active electrodes at the following scalp sites: Fp1, Fp2, F7, F3, Fz, F4, F8, FC5, FC1, FC2, FC6, T7, C3, Cz, C4, T8, CP5, CP1, CP2, CP6, P7, P3, Pz, P4, P8, O1, O2, and on the right mastoid. The ground electrode was placed at AFz and the data were online referenced to the left mastoid. Four additional electrodes attached to the two external canthi, and above and below the right eye, monitored the eye movements (EOG). The EEG and EOG were sampled with 500 Hz and online bandpass filtered between 0.01 and 250 Hz, with half-power and a corner frequency of 3 dB.

### Data preprocessing and analysis

The EEG data were preprocessed and analyzed in MATLAB 2011b (The Math Works, Inc., M.A.) using the EEGlab toolbox (Delorme and Makeig, 2004) and the ERPlab toolbox extensions (http://erpinfo.org/erplab). Statistics were performed in SPSS 17.0 (SPSS Inc., IL).

EEG measurements were digitally-filtered offline using a non-causal Kaiser-windowed sinc Finite Impulse Response filter with a half-amplitude (−6 dB) bandpass of 0.1–35 Hz, a Kaiser-window beta of 5.65326, ripple of 0.001, a transition band of 0.5 Hz, and an order of 3624 (Widmann, 2006; Widmann and Schröger, 2012). The four vertical and horizontal ocular channels were bipolarized into vertical and horizontal EOG. Furthermore, the data were re-referenced to the linked mastoids for all analyses except for mastoid amplitudes, for which data were re-referenced to common average. Epochs were created from −100 to 500 ms for the oddball paradigm and from −200 to 1000 ms for the semantic paradigms. Time windows from −100 to 0 ms for the oddball paradigm and −200 to 0 for the semantic paradigms were used as a baseline. Eye movement artifacts were corrected using a regression-based procedure (Gratton et al., 1983) and all trials containing signal changes of ±80 μV were excluded from further analysis using an automatic peak-to-peak detection method. Before entering the statistical analyses, all trials containing key presses in the ignore task and all misclassified sentences and word pairs as well as missed deviants in the focused task were discarded. Finally, grand averages were obtained. For the statistical analyses, we selected the electrodes F3, Fz, F4, C3, Cz, C4, P3, Pz, and P4. The relevant time windows for the component analyses in all the paradigms were defined by visual inspection. In the oddball paradigm, visual analysis yielded a large negative component with two peaks in the passive and focused task. Thus, two time windows ranging from 110 to 170 ms and 170 to 230 ms were used for the analyses. In the semantic paradigms, the time windows were set to 300–600 ms for the word priming paradigm and 250–650 ms for the sentence paradigm. We also obtained difference waves by subtracting the standards from the deviants (oddball paradigm), the related word pairs from the unrelated word pairs (word priming paradigm), and the correct sentences from the incorrect sentences (sentence paradigm). The mean amplitude under the curve in the corresponding time windows entered the statistical analyses (repeated-measures ANOVA). ANOVAs were calculated for each paradigm separately and for all results, we report the Greenhouse-Geisser corrected values (Greenhouse and Geisser, 1959) since the assumption of sphericity was violated in all the analyses and values of epsilon were smaller than 0.75, which is the recommended threshold for application of the Greenhouse-Geisser correction (Girden, 1992). Planned *t*-tests were conducted to detect any significant differences between individual tasks as defined in our hypotheses. In addition, we report the effect size partial eta squared (η^2^_*p*_) for all main effects of task and the effect size Cohen's *d* for planned *t*-tests. According to Cohen (1988), η^2^_*p*_ = 0.01 or *d* = 0.2 represents a small effect, η^2^_*p*_ = 0.06 or *d* = 0.5 a medium effect and η^2^_*p*_ = 0.14 or *d* = 0.8 a large effect.

In addition, we calculated the voltages and scalp current densities (SCDs) maps for each time window of interest. The scalp current densities were derived from the voltage distribution using a spherical spline surface Laplacian algorithm (Perrin et al., 1989). The Laplacian had a conductivity of 0.45 Siemens/m and was conducted by using the second derivative of the potential distribution. We set the maximum degree of the Legendre polynomials to 50 and the order of splines to 4.

## Results

### Subjective effort and performance

The participants indicated their subjective effort after the completion of each paradigm in each task (Table 1). We performed a repeated-measures ANOVA with the two factors task (ignore, passive, focused) and paradigm (word priming, sentences, oddball) to detect any significant variation as a function of the attentional instruction and auditory stimuli.

**Table 1:** **Mean values (*M*) and standard deviations (*SD*) of subjective effort listed for all three paradigms (columns) and tasks (rows)**.

		**Word-pairs**	**Sentences**	**Oddball**	**Mean per task**
Ignore task	*M*	32.55	55.99	46.89	45.15
	*SD*	22.08	56.99	40.52	42.66
Passive task	*M*	72.91	80.01	89.39	80.77
	*SD*	47.24	58.11	55.82	53.34
Focused task	*M*	73.76	85.98	83.71	81.15
	*SD*	46.09	59.84	54.37	52.98

Scale range: 0–220, seven labels ranging from “rarely strenuous” at 20 to “extraordinarily strenuous” at about 205.

Subjectively experienced effort varied significantly according to the task [*F*_(2, 34)_ = 9.93, *p* = 0.001, η^2^_*p*_ = 0.369) with the passive and focused tasks being judged as more effortful than the ignore task (see Table 1). The main effect of paradigm was not significant. This result partially confirms our assumption since the ignore task was least effortful, but the passive and focused tasks were judged as equally demanding.

The ignore task required the participants to press a button in response to a predefined scene in a silent movie. Eleven participants (61.1%) detected all the scenes in one of the three paradigms, six participants (33.3%) detected all the scenes in two paradigms, and one participant (5.6%) correctly detected all the scenes in all of the paradigms. The number of missed scenes ranged between one and four. Three participants indicated the scene although it had not appeared. Significantly more scenes were missed in the sentence paradigm (*M* = −1.05) than in the word priming (*M* = 0.27), or oddball paradigms (*M* = 0.39): *F*_(2, 34)_ = 4.38, *p* = 0.022, η^2^_*p*_ = 0.203.

Table 2 presents the number of correct and incorrect detections as well as misses in the three paradigms in the focused task. In the oddball and sentence paradigms, two participants were excluded because their results differed from the group mean by more than two standard deviations.

**Table 2:** **Amount of correct and incorrect answers and misses for the focused attention task for all paradigms, all values are listed in percent (%)**.

		**Deviant detection/correct answers**	**Wrong detections/incorrect answers or false alarms**	**Misses**
Oddball paradigm	*M*	94.24	5.76	5.59
Deviant detection	*SD*	6.22	6.22	4.82
Word-pairs	*M*	95.33	4.17	0.5
Related	*SD*	3.12	3.09	1.04
Word-pairs	*M*	93.00	4.83	2.17
Unrelated	*SD*	5.58	3.26	2.97
Sentences	*M*	82.82	12.47	4.35
Correct	*SD*	8.44	1.28	7.91
Sentences	*M*	80.47	13.53	6.00
Incorrect	*SD*	7.65	1.55	7.71

For each of the two answer categories (correct and incorrect), we performed a repeated-measures ANOVA including the factors paradigm (word priming vs. sentences) and stimuli (related/correct vs. unrelated/incorrect). The ANOVA for correct answers yielded a significant main effect of paradigm [*F*_(1, 15)_ = 154.88, *p* < 0.001, η^2^_*p*_ = 0.912] and stimuli [*F*_(1, 15)_ = 7.10, *p* = 0.018, η^2^_*p*_ = 0.321], indicating that the participants identified more related word pairs as related than correct sentences as correct and in general made less errors detecting related word pairs and correct sentences than detecting unrelated word pairs and incorrect sentences. The ANOVA for incorrect answers confirmed this result by revealing a main effect of paradigm [*F*_(1, 15)_ = 279.57, *p* < 0.001, η^2^_*p*_ = 0.949], indicating that generally more errors occurred in the sentence paradigm.

### Physiological data

To analyze the ERP data, we performed multivariate ANOVAs with repeated-measures with the difference waves as dependent variables including the following three factors: task (ignore, passive, focused), region (frontal, central, parietal), and laterality (left, middle, right). Region and task were included in the analysis to also describe the spatial distribution of ERP components. Furthermore, *t*-tests comparing the mean amplitudes to zero were conducted for midline electrode sites to detect the elicitation of a component.

#### ERP results of the oddball paradigm

A *t*-test for the difference from zero demonstrated large and significant negative components in all three tasks at the electrode sites Fz, Cz, and Pz in both time windows (all *p* < 0.001). The first deflection peaking at about 150 ms was interpreted as a late component of N1 (Luck, 2005), which is also sensitive to attention (Woldorff et al., 1993). The second peak at about 190 ms was interpreted as an MMN (Luck, 2005). For both responses, we found the typical reversal of polarity at the mastoid electrodes in all three tasks.

The repeated-measures ANOVA revealed a main effect of task on the early negative deflection [*F*_(2, 34)_ = 7.19, *p* = 0.003, η^2^_*p*_ = 0.297] and the late negative deflection [*F*_(2, 34)_ = 11.80, *p* = 0.001, η^2^_*p*_ = 0.410]. In both cases, the deviant-minus-standard difference was largest in the focused task (*M*_*N*100_ = −4.87 μV, *M*_MMN_ = −4.12 μV), smaller in the passive task (*M*_*N*100_ = −4.04 μV, *M*_MMN_ = −2.99 μV), and smallest in the ignore task (*M*_N100_ = −3.67 μV, *M*_MMN_ = −1.85).

**Figure 1:**
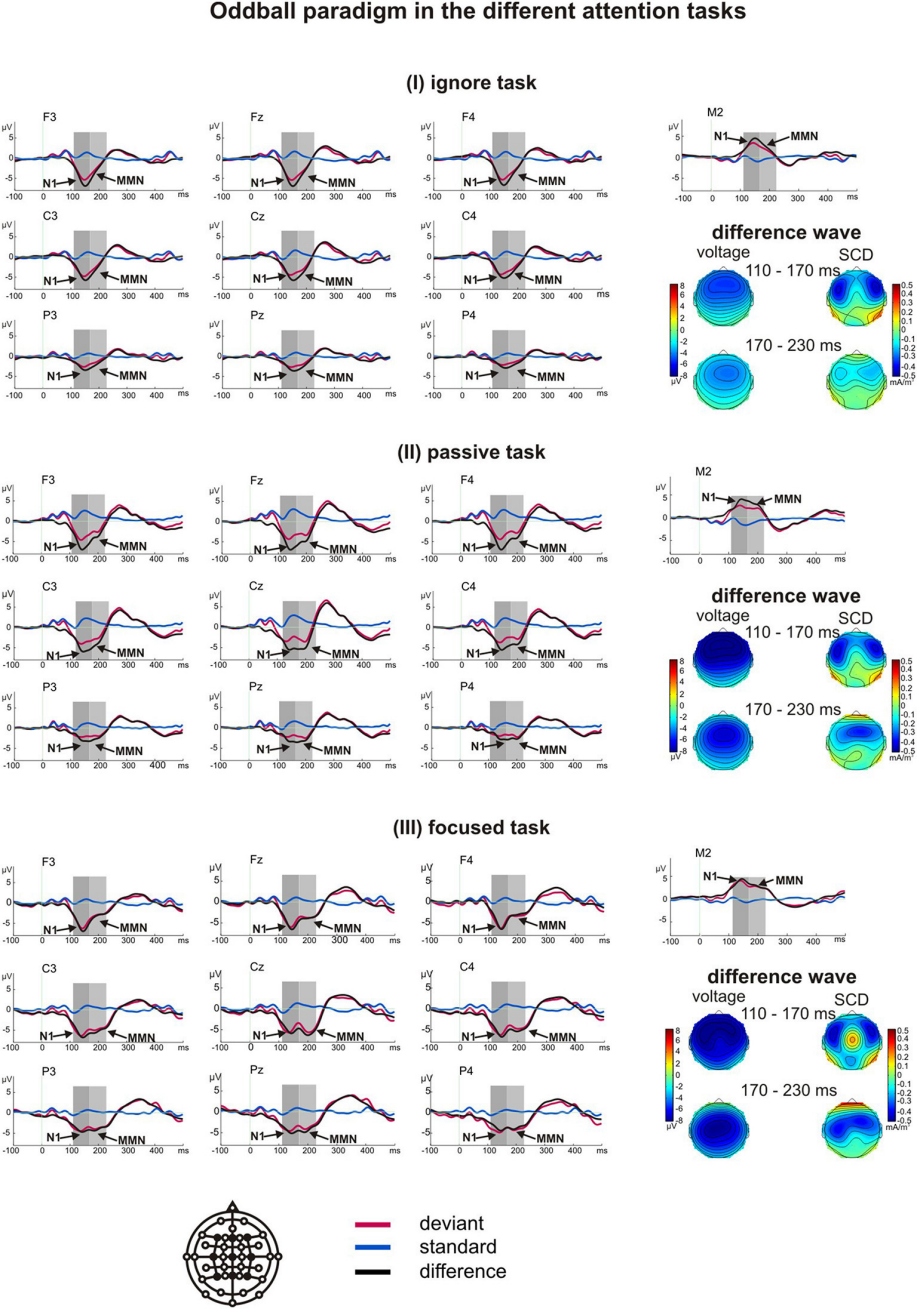
**Grand average ERPs of the oddball paradigm obtained in the ignore task (I), passive task (II) and focused task (III)**. The arrows indicate the critical significant components N1 and MMN and the gray boxes represent the time window used for analysis. The voltage and scalp current density (SCD) maps of the difference waves for both time windows reveal the respective scalp distributions and the possible generators for the components.

Planned *t*-tests revealed significant differences over central and parietal regions between the passive and focused task (except Cz, all *t* > 2.56, all *p* < 0.020, all *d* > 0.52), and between the ignore and focused task (except Cz, all *t* > 2.95, all *p* < 0.009, all *d* > 0.70) for the N1. The passive and ignore task did not differ significantly in any region. MMN amplitudes differed over all regions between the ignore and passive task (except P4, all *t* > 3.00, all *p* < 0.008, all *d* > 0.59), and between the ignore and focused task (all *t* > 2.26, all *p* < 0.037, all *d* > 0.52; F3 and F4 showed a trend with both *t* > 1.96, *p* < 0.066, *d* > 0.45). Significant differences over central and parietal regions were also found between the passive and focused task (all *t* > 2.12, all *p* < 0.049, all *d* > 0.50). Additional results are listed in Table 3. All main effects and interactions reached levels of significance. These results and the voltage and SCD maps demonstrated a fronto-central distribution of the N1 expanding over left parietal areas (Figure 1). The MMN also originated over fronto-central areas and was largest over the central scalp in the focused task.

**Table 3:** **Oddball paradigm**.

		**(a)**	**(b)**	**(c)**	**(d)**
Task	*F*	719	11.80	5.37	7.73
	*df*	2.34	2.34	2.20	2.20
	*p*	0.003^**^	0.001^**^	0.016^*^	0.008^**^
	η^2^_*p*_	0.297	0.410	0.349	0.436
Region	*F*	51.16	9.39	37.67	16.80
	*df*	2.34	2.34	2.20	2.20
	*p*	0.000^***^	0.001^**^	0.000^***^	0.001^**^
	η^2^_*p*_	0.751	0.356	0.790	6.27
Laterality	*F*	40.45	14.08	21.80	15.80
	*df*	2.34	2.34	2.20	2.20
	*p*	0.000^***^	0.000^***^	0.000^***^	0.000^***^
	η^2^_*p*_	0.704	0.453	0.686	0.612
Task*region	*F*	13.60	8.83	4.81	3.74
	*df*	4.68	4.68	4.40	4.40
	*p*	0.000^***^	0.001^**^	0.027^*^	0.045^*^
	η^2^_*p*_	0.445	0.342	0.325	0.272
Task*laterality	*F*	6.05	4.68	3.00	2.87
	*df*	4.68	4.68	4.40	4.40
	*p*	0.008^**^	0.019^*^	0.086	0.095
	η^2^_*p*_	0.263	0.216	0.231	0.223
Region*laterality	*F*	53.06	30.32	30.33	24.04
	*df*	4.68	4.68	4.40	4.40
	*p*	0.000^***^	0.000^***^	0.000^***^	0.000^***^
	η^2^_*p*_	0.757	0.641	0.752	0.706
Task*region*laterality	*F*	10. 84	6.32	7.07	4.64
	*df*	8.136	8.136	8.80	8.80
	*p*	0.000^***^	0.007^**^	0.007^**^	0.035^*^
	η^2^_*p*_	0.389	0.271	0.414	0.317

Results of the repeated measures ANOVA including the factors: task (ignore, passive, focused), region (frontal, central, parietal) and laterality (left, middle, right); including all subjects (a) N100 time-window (110–170 ms) and (b) MMN time-window (170–230 ms). Results of the same repeated measures ANOVA for the subgroup of 11 participants (c) N100 time window (110–170 ms) and (b) MMN time-window (170–230 ms).

* p < 0.05,

** p < 0.01,

*** p < 0.001.

Additional analyses were conducted to support the assumption of two distinct potentials. We calculated a repeated-measures ANOVA including the factors time window (110–170 ms, 170–230 ms), task (ignore, passive, focused), and region (midline electrodes Fz, Cz, and Pz). The main effect of time window showed a trend [*F*_(1, 17)_ = 4.21, *p* = 0.056, η^2^_*p*_ = 0.198] indicating higher amplitudes for N100 (*M*_N100_= −4.29, *M*_MMN_ = −3.31). Importantly, we found a significant main effect of task [*F*_(2, 34)_ = 10.32, *p* = 0.002, η^2^_*p*_ = 0.378] indicating amplitudes to be lowest in the ignore task, higher in the passive task, and highest in the focused task. The significant interaction of time window and task [*F*_(2, 34)_ = 4.84, *p* = 0.021, η^2^_*p*_= 0.222] indicates a differential influence of task on the amplitudes in the two time windows. *Post-hoc* comparisons revealed that the ignore and passive task only differed in the MMN time window (*p* = 0.002, *d* = 0.84), the ignore and focused task differed in both time windows with a larger effect for MMN (*p*_N100_ = 0.021, *d*_N100_ = 0.62, *p*_MMN_ = 0.001, *d*_MMN_ = 1.11), and that the passive and focused task showed a trend to differ from each other (*p*_N100_ = 0.081, *d*_N100_ = 0.39, *p*_MMN_ = 0.071, *d*_MMN_ = 0.45).

To support the notion of MMN amplitudes varying as a function of task, we also calculated an ANOVA over amplitudes at the right mastoid including the factor task (ignore, passive, focused). Mastoid amplitudes in the relevant time windows are also depicted in Figure 1. The deflections of positive polarity at the mastoid amplitudes in the time window of 170–230 ms differed significantly according to the task [*F*_(2, 34)_ = 10.69, *p* = 0.000, η^2^_*p*_ = 0.386] with the amplitude being largest in the focused task (*M*_focused_ = 2.76), smaller in the passive task (*M*_passive_ = 2.42), and smallest in the ignore task (*M*_ignore_ = 1.42). Planned *t*-tests revealed significant differences between the ignore and passive task (*t* = 3.68, *p* = 0.002, *d* = 0.85), and between the ignore and focused task (*t* = 4.38, *p* = 0.000, *d* = 0.94).

Also the amplitudes recorded at the mastoid exhibited differential effects in the two time windows (110–170 ms, 170–230 ms). A main effect of time window [*F*_(1, 17)_ = 11.13, *p* = 0.004, η^2^_*p*_ = 0.396] indicated higher amplitudes for N100 than the MMN (*M*_N100_= −3.41, *M*_MMN_ = −2.20), and a main effect of task [*F*_(2, 34)_ = 7.49, *p* = 0.002, η^2^_*p*_ = 0.306] indicated amplitudes to be lowest in the ignore task, higher in the passive task, and highest in the focused task. The significant interaction between time window and task [*F*_(2, 34)_ = 3.85, *p* = 0.048, η^2^_*p*_= 0.185] indicated a smaller amplitude in the ignore task, as compared with both, passive and focused tasks, in the MMN time window (*p* < 0.002, *d* > 0.85), whereas the passive and focused task showed a trend to differ in the N100 time window (*p* = 0.068, *d* = 0.34).

**Figure 2:**
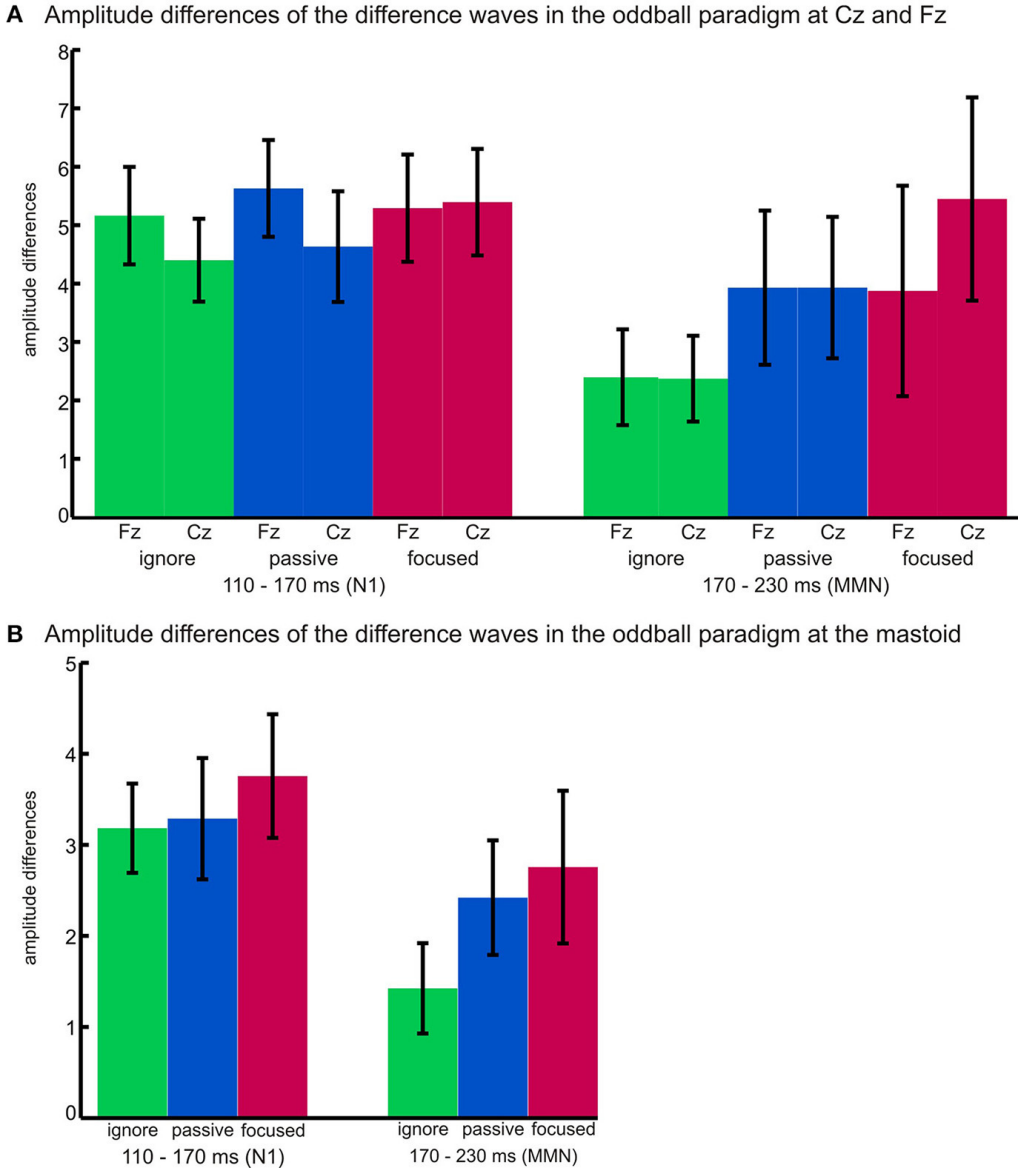
**MMN amplitude differences at (A) Fz and Cz and (B) the right mastoid for each task (ignore, passive, focused) in the two time windows (110–170 ms and 170–230 ms)**. Error bars represent standard deviations.

Figure 2 illustrates the differential effects of attention within the two time windows and depicts the absolute values of amplitude differences at Fz and Cz (a) as well as the amplitudes at the right mastoid (b) for each task (ignore, passive, focused) in both time windows (N1: 110–170 ms and MMN: 170–230 ms).

***Oddball paradigm: Subgroup analysis for the ignore task.*** The difference wave in the ignore task revealed only one distinct negative curve while a double peak emerged in the focused and passive tasks. To evaluate the waveform elicited in the ignore task, we visually inspected the ERP curves of all the participants individually. We found a double peak (N1 and MMN) in 11 of the 18 participants. These 11 participants did not differ from the others in terms of age, sex, time of measurement, or order of tasks. A statistical analysis for these 11 participants resulted in a main effect of task for the early negative deflection [*F*_(2, 20)_ = 5.37, *p* = 0.016, η^2^_*p*_ = 0.349], and the late negative deflection [*F*_(2, 20)_ = 7.73, *p* = 0.008, η^2^_*p*_ = 0.436] with the deviant-minus-standard difference being largest in the focused task (*M*_*N*100_ = −5.14 μV, *M_MMN_* = −4.94 μV), smaller in the passive task (*M*_*N*100_ = −3.97 μV, *M_MMN_* = −3.51 μV), and smallest in the ignore task (*M*_*N*100_ = −3.66 μV, *M_MMN_* = −2.23 μV). Thus, the results for these 11 participants did not differ from those of all 18 participants. Further significant results are depicted in Table 3. Again, all main effects and interactions except for task^*^laterality reached significance. These results reveal a fronto-central distribution of the N1 and MMN with both expanding over left parietal regions.

Planned *t*-tests for these 11 participants revealed that the N1 amplitudes differed significantly between the ignore and focused task at central and parietal sites (except Cz, all *t* > 2.50, all *p* < 0.031, all *d* > 0.74), and between the passive and focused task over parietal sites and at C4 (all *t* > 2.99, all *p* < 0.013, all *d* > 1.12). No significant difference was found between the passive and ignore task. MMN amplitudes differed significantly at all sites between the ignore and focused task (all *t* > 2.72, all *p* < 0.022, all *d* > 1.00), and between the ignore and passive task (except P4, all *t* > 2.35, all *p* < 0.040, all *d* > 1.02). No significant differences were found between the passive and focused task.

Taken together, in the oddball paradigm the MMN was found to be largest in the focused and passive task and smaller in the ignore task. This result was the same in the whole sample of 18 participants, and in the subsample of 11 participants having two distinct peaks.

#### Results of the word priming paradigm

**Figure 3:**
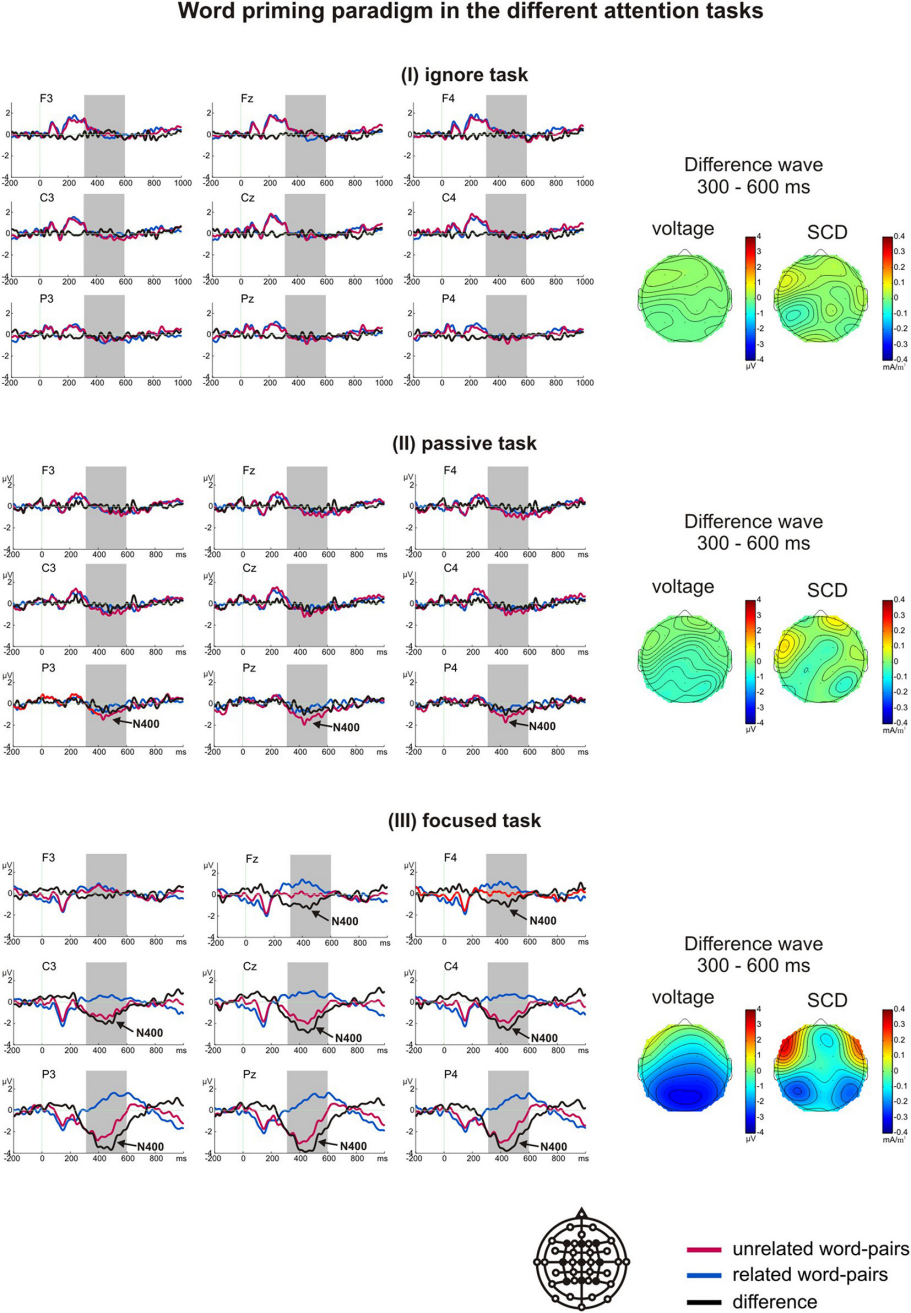
**Grand average ERPs of the word priming paradigm in the ignore task (I), passive task (II) and focused task (III)**. The arrows indicate whether the N400 was significantly different from zero within the analyzed time window (marked by gray boxes). The voltage and scalp current density (SCD) maps of the difference waves for the N400 time window depict the scalp distributions and the possible generators.

A *t*-test for difference from zero indicated a significant N400 at Pz in the passive task (*p* = 0.02) and at Fz, Cz, and Pz in the focused task (*p* < 0.001). No N400 was observed in the ignore task. In the repeated-measures ANOVA, we found a significant main effect of task [*F*_(2, 34)_ = 35.853, *p* =0.004, η^2^_*p*_ = 0.678] with the N400 being largest in the focused task (*M* = −1.64 μV), smaller in the passive task (*M* = −0.26 μV), and virtually absent (*M* = −0.06 μV) in the ignore task (Figure 3). Table 4 depicts the further results of the main effects of laterality and region as well as the interactions.

**Table 4:** **Results of the repeated ANOVA including the factors: task (ignore, passive, focused), region (frontal, central, parietal) and laterality (left, middle, right), within the N400 time range, of the (a) word priming paradigm and of the (b) sentences paradigm**.

		**(a)**	**(b)**
Task	*F*	35.85	13.52
	*df*	2.34	2.34
	*p*	0.000^***^	0.000^***^
	η^2^_*p*_	0.678	0.458
Region	*F*	38.14	16.63
	*df*	2.34	2.34
	*p*	0.000^***^	0.000^***^
	η^2^_*p*_	0.692	0.510
Laterality	*F*	5.59	4.46
	*df*	2.34	2.34
	*p*	0.019^*^	0.037^*^
	η^2^_*p*_	0.247	0.218
Task*region	*F*	17.25	9.29
	*df*	4.68	4.68
	*p*	0.000^***^	0.000^***^
	η^2^_*p*_	0.504	0.367
Task*laterality	*F*	4.46	3.68
	*df*	4.68	4.68
	*p*	0.012^*^	0.033^*^
	η^2^_*p*_	0.208	0.187
Region*laterality	*F*	8.05	10.22
	*df*	4.68	4.68
	*p*	0.003^**^	0.001^**^
	η^2^_*p*_	0.321	0.390
Task*region*laterality	*F*	8.89	4.83
	*df*	8.136	8.136
	*p*	0.000^***^	0.007^**^
	η^2^_*p*_	0.343	0.232

* p < 0.05,

** p < 0.01,

*** p < 0.001.

Planned *t*-tests revealed significant differences at all sites between the ignore and focused task (except F3, all *t* > 2.26, all *p* < 0.037, all *d* > 0.93), and between the passive and focused task (except F3, all *t* > 2.26, all *p* < 0.037, all *d* > 0.77). The ignore and passive task did not differ significantly. All main effects and interactions were significant. The results of the ANOVA and the voltage and SCD maps revealed the largest N400 over left parietal regions, expanding to central regions in the focused task.

In addition to the N400 component, noticeable deflections could be seen in several time windows. To test whether these deflections reflect different brain processes in reaction to related and unrelated word pairs, separate repeated-measures ANOVAs, including the factors task, region, and laterality, were extended by the factor stimulus (related vs. unrelated): In the focused task a positivity occurred between 0 and 190 ms, but related and unrelated second words did not differ significantly [*F*_(1, 17)_ = 3.40, *p* = 0.08]. In the passive task a positivity occurred between 110 and 160 ms. Again, related and unrelated second words did not differ significantly [*F*_(1, 17)_ = 0.62, *p* = 0.441]. In the ignore and passive task, two positivities ranging from 40 to 100 ms and 170 to 300 ms were elicited. Again, related and unrelated second words did not differ significantly in either time window [40–100 ms: *F*_(1, 17)_ = 0.76, *p* = 0.787 for the main effect of stimulus, *F*_(1, 17)_ = 0.32, *p* = 0.581 for the interaction task^*^stimulus; 170 to 300 ms: *F*_(1, 17)_ = 0.00, *p* = 0.995 for the main effect of stimulus, *F*_(1, 17)_ = 0.26, *p* = 0.128 for the interaction task^*^stimulus].

Taken together, the N400 was the only component that reflected different processes in reaction to related and unrelated word pairs. In addition, the results for the word priming paradigm revealed the largest N400 effect in the focused task, a smaller one in the passive task and no N400 in the ignore task.

#### Results of the sentence paradigm

**Figure 4:**
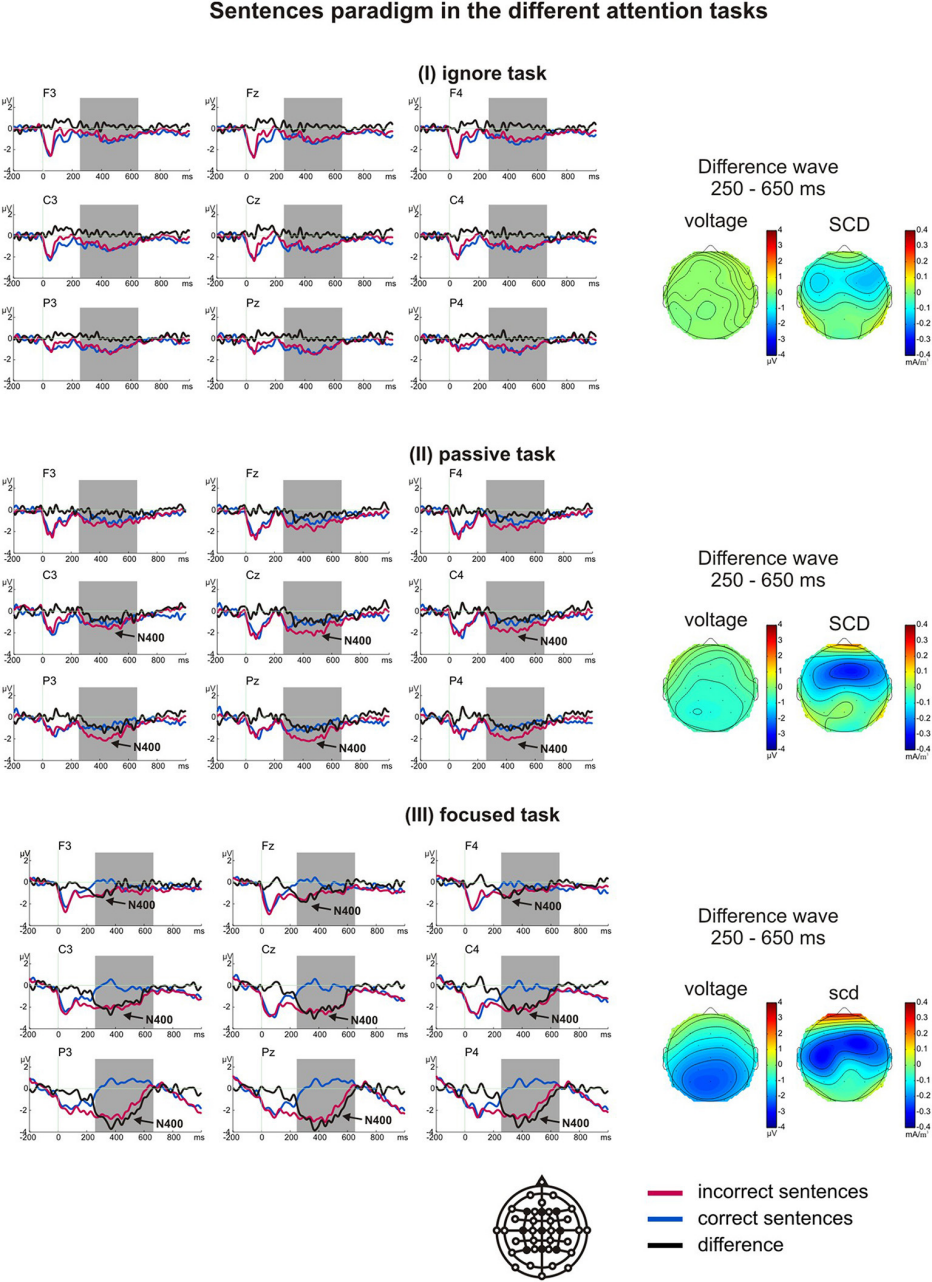
**Grand average ERPs of the sentence paradigm in the ignore task (I), passive task (II) and focused task (III)**. The arrows indicate whether the N400 difference was significantly different from zero within the analyzed time window (marked by gray boxes). The voltage and scalp current density (SCD) maps of the difference waves for the N400 time window depict the scalp distributions and the possible generators.

In the sentence paradigm, one participant's data were excluded due to technical problems during recording. A *t*-test for difference from zero showed an N400 component in the passive task (only at Cz and Pz with *p* < 0.021) and the focused task (*p* < 0.016). No N400 component was elicited in the ignore task. A significant main effect of task on the N400 effect was evident [*F*_(2, 34)_ = 13.52, *p* = 0.000, η^2^_*p*_ = 0.458], with the difference being largest in the focused task (*M* = −1.52 μV), strongly attenuated in the passive task (*M* = −0.57 μV), and not significantly different from zero (*M* = −0.12 μV) in the ignore task (Figure 4).

Planned *t*-tests revealed significant differences at central and parietal sites between the passive and focused task (all *t* > 2.47, all *p* < 0.025, all *d* > 1.64), and between the ignore and passive task (all *t* > 2.56, all *p* < 0.021, all *d* > 0.77). The ignore and focused task differed significantly at all sites (all *t* > 3.42, all *p* < 0.003, all *d* > 0.74). All main effects and interactions were significant (Table 4). Like in the word priming paradigm, in the focused task the N400 was largest over left parietal regions expanding over central regions.

In addition to the N400 component, a negativity could be observed between 0 and 170 ms in all three tasks. This negativity did not distinguish between correct and incorrect sentence endings [*F*_(1, 16)_ = 0.02, *p* = 0.898 for the main effect of stimulus, *F*_(2, 32)_ = 1.73, *p* = 0.193 for the interaction task^*^stimulus]. This component was interpreted as a continuing negativity in response to the previous words of the sentences.

Taken together, in the sentence paradigm the N400 was the only component reflecting different brain processes in response to correct and incorrect sentences. We found the largest N400 effect in the focused task, a smaller one in the passive task and no N400 in the ignore task.

## Discussion

The goal of the present study was to investigate the effect of different attentional instructions on ERP measures. Firstly, all the ERP effects described in the corresponding literature (MMN, single word N400, sentence N400) were successfully replicated. Secondly, it was shown that these effects significantly depended on the direction of attention. The MMN was larger in the focused and passive task compared to the ignore task, thus also indicating an attentional effect on the supratemporal component of the MNN. The N400 successively declined with decreasing attention and was extinct in the ignore task. The subjective effort was equally high in the focused and passive tasks, and much lower in the ignore task. Thus, our hypotheses concerning ERPs were fully confirmed, those for subjective effort partially.

**Figure 5:**
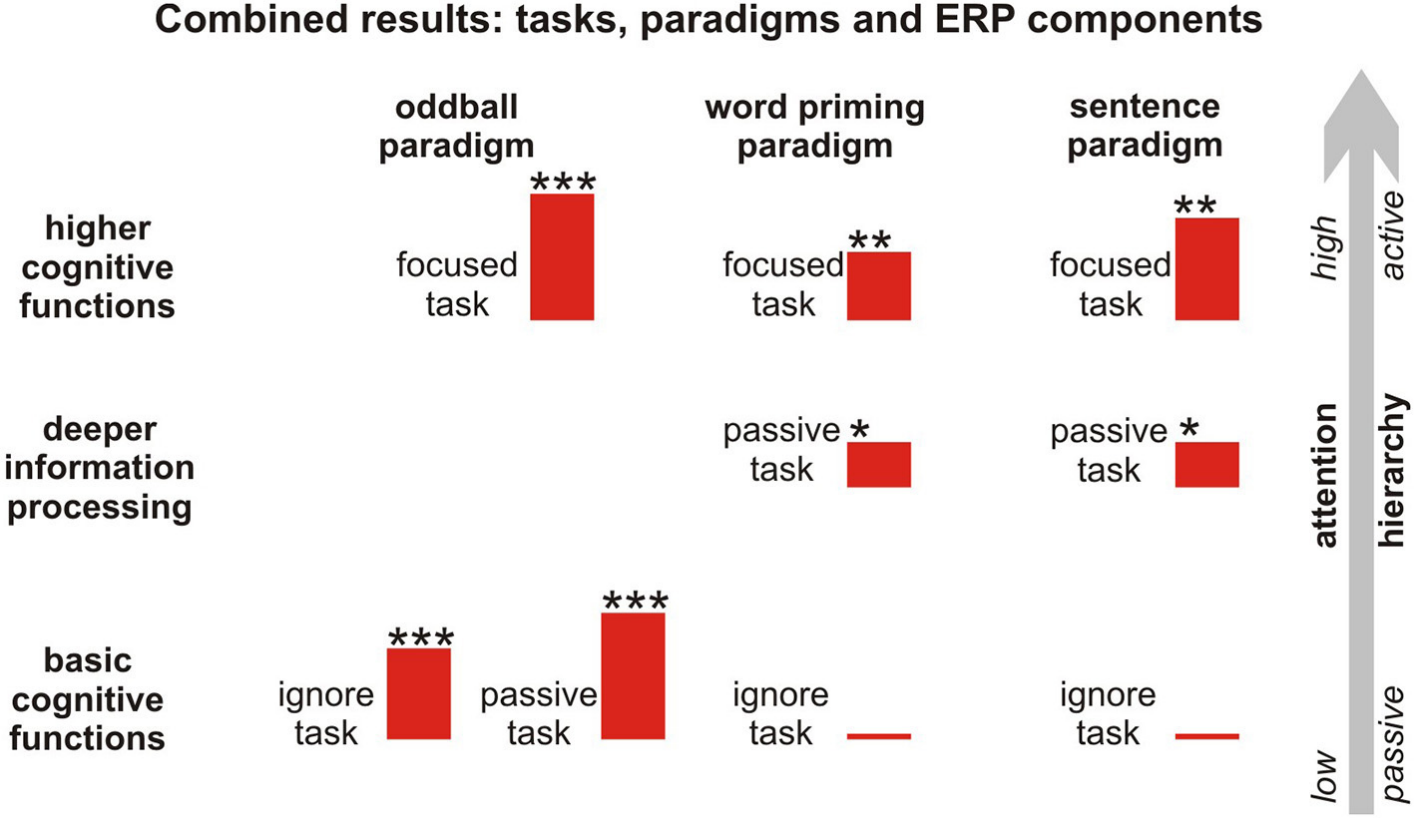
**Hierarchical approach to information processing including all attentional tasks, paradigms, and respective components**. The red bars reflect the mean amplitude of the difference curves of the respective component, whereas ^*^ represents the number of regions (frontal, central, parietal) showing a significant MMN/N400 effect. ^**,***^p < 0.1.

We reported the ERP results in each paradigm in relation to each individual task. However, in order to be able to judge the residual cognitive functions in patients, it is necessary to include different paradigms that investigate various cognitive processing steps and instructions (passive and active). These paradigms are commonly constructed in line with a so-called hierarchical approach (Kotchoubey et al., 2005; Owen et al., 2005; Kübler and Kotchoubey, 2007), whereby the difficulty of the tasks is gradually increased from passive, easy tasks to active, more difficult ones. In Figure 5, the results of the tasks and paradigms are depicted according to the hierarchical approach. These results suggest that attention toward a stimulus enhances the specific components, especially in the language paradigms eliciting the N400 component, irrespective of whether the N400 is elicited by word pairs or sentences.

In the following section we discuss the applied multicomponent approach including the three attentional tasks and the respective ERP components N400 and MMN, as well as the subjective effort. Alongside, factors limiting the scope of our findings are discussed and finally, a conclusion regarding attentional instructions, especially in relation to patient measurements, is drawn.

A comparison between the different paradigms and tasks revealed similar, but slightly different effects of attention for MMN and N400. The MMN, representing basic cognitive functions, exhibited higher amplitudes in the passive and focused tasks and was reduced, but not extinct, in the ignore task. The N400, representing higher cognitive functions, gradually decreased with decreasing attention from to focused to the passive task and was completely absent in the ignore task. These results not only confirm the expected variation according to attention allocation theories, but also highlight the importance of appropriate instructions in ERP measurements, especially in semantic paradigms designed to elicit an N400. If ERPs are recorded in DoC patients to detect basic or higher cognitive functions, passive instructions might lead to attenuated potentials, which are difficult to interpret, and thus to misjudgment of the patients' cognitive capacity. A positive finding in a passive version of a language paradigm unequivocally indicates the patient's ability of semantic processing, but a negative finding remains ambiguous, because it can result not only from the real lack of semantic competence but also from many other factors such as a low arousal level.

In line with our hypothesis, an N400 component was elicited in both semantic paradigms and was affected by attentional modulation. Thus, we found the largest N400 component in the focused task and a diminished one in the passive task. No N400 was found in the ignore task. These findings support the N400 as a potential to detect higher cognitive functions that require directed attention and confirm previous studies showing strong attenuation or extinction of the N400 effect when attention is not directed toward the stimuli (McCarthy and Nobre, 1993; Chwilla et al., 1995). However, other studies did not find that N400 is modulated by the depth of processing level (Connolly, 1990; Relander et al., 2009). In their review, Deacon and Shelley-Tremblay (2000) concluded that the N400 does not necessarily require attention but only occurs if the processing of the stimuli is not actively inhibited. This view is supported by the present data insofar that active inhibition in the ignore task led to extinction of the N400 potential. In the passive task, where no specific tasks had to be performed apart from mere listening, the N400 was strongly attenuated but still present.

Also Kutas and Federmeier (2011) described in their review that the N400 incorporates characteristics of both, automatic and controlled processing. Daltrozzo and colleagues came to the conclusion that sentence N400 is mainly a reflection of controlled processes, although they did not rule out that single word N400 might also include automatic aspects (Daltrozzo et al., 2012). The present results tend to support the view that both single word and sentence N400 manifest controlled processes: A pronounced N400 component was only evident when attention was focused on the verbal stimuli. However, an external behavioral measure of the level of processing was only applied in the focused task. The passive and ignore tasks did not demand a behavioral reaction to the auditory stimuli but rather caused distraction (ignore task) or required behavioral passiveness (passive task). Behavioral information is similarly lacking when ERPs are used in the clinical assessment of DoC and other non- and low-responsive patients.

While the N400 in the semantic paradigms was strongly attenuated in the passive task or even absent in the ignore task, a significant MMN was elicited at all levels of attention, with attention being directed away, passive, or highly focused on the auditory stimuli. Even though there is still debate about the possible effect of voluntary attention on the MMN, we hypothesized that the MMN would also be modulated by attention because of the standard formation process postulated by Sussman (2007). Accordingly, the MMN amplitude was affected by instructions: the amplitudes were largest in the focused and passive task and smaller in the ignore task. However, the interpretation of these findings is complicated by a potential overlap of the MMN with N1 and N2b, which will be both discussed in the following.

In an oddball paradigm, the MMN might be overlapped by an N1. In the grand average of the ignore task, we found only one distinct peak at about 150 ms which, following our earlier interpretation, represents an N1. However, an analysis on the single subject level revealed a second peak at about 190 ms in 11 of the 18 participants, indicating that an MMN overlapped with the N1. Five observations support this hypothesis: Firstly, in the grand average of all participants, we also observed a trend toward a second peak at central electrodes. Secondly, the N1 difference curves peaked slightly later in the ignore task (around 155 ms) than in the passive and focused tasks (around 145 ms). Attentional processes can affect the N1, particularly with short inter-stimulus intervals (Schwent et al., 1976; Woldorff et al., 1993). Thirdly, the MMN peaks slightly earlier in less demanding conditions involving the visual modality (Rissling et al., 2013), e.g., like in our ignore task. These shifts of N1 and MMN toward each other might have contributed to the overlap, which is common and renders them difficult to differentiate (Näätänen and Picton, 1987). Fourthly, the single peak observed in the ignore task exhibited a distinctly higher amplitude than each of the two peaks in the other tasks, which also supports the existence of an overlap. Finally, our results revealed a differential effect of task on the amplitudes recorded within the N100 vs. the MMN time range, over scalp electrodes and at the mastoid. Taken together, these findings indicate relative independence and a partial functional dissociation between MMN and N1.

Even though the MMN and N1 are regarded as spatiotemporally distinct (e.g., Campbell et al., 2007), separating them in the present experiment is problematic. Following the MMN-N1 additivity hypothesis (Campbell et al., 2007), differentiating and eliminating the N1 effect from the MMN would have required a second control condition, in which the deviant tones are presented in a sequence of many other tones to control for the frequency specific refractoriness. By using such a control condition, it is possible to differentiate the processing of deviant and control tones as reflected by N1 and MMN amplitudes using the components' distributions in the N1/MMN latency range. The negative ERP amplitudes following deviant tones would be larger at fronto-central sites than following the physically identical tones used in the control condition. Thus, this comparison differentiates the memory-based and refractoriness effect of the N1 and MMN (Schröger and Wolff, 1996; Jacobsen and Schröger, 2001). We did not include such a separate condition because we used a short paradigm which has already proven to be applicable in DoC patients (Kotchoubey et al., 2005).

Besides an overlap of MMN with N1, MMN may also overlap with N2b under certain conditions (Sussman, 2007). Even though both, MMN and N2b, are elicited by rare events in an oddball paradigm, it is assumed that the MMN is elicited by both attended and unattended stimuli, whereas the N2b only occurs when attention is directed to target stimuli (Muller-Gass et al., 2005; Folstein and Van Petten, 2007). Thus, in most MMN studies, attention is directed to irrelevant stimuli in order to avoid an N2b. In the current study, an N2b can only be ruled out for the ignore task, and may have been elicited in the passive and focused tasks since the participants may have concentrated on the deviant stimuli in the passive task and were directly instructed to do so in the focused task. Thus, attention to the stimuli might have added an N2b effect to the existing MMN rendering them difficult to discriminate. Also the SCD data do not allow for disentangling the potential overlap because they revealed that the sources are located in the fronto-central regions in both time windows. Nevertheless, the negative deflection in the passive and focused tasks exhibited a positive ratio over frontal areas, which might reflect the overlap of the MMN and N2b. This positive ratio was lacking in the SCD of the earlier time range in the ignore task. In addition, the SCD was similar to the MMN reported by Pieszek et al. (2013). Significant differences in amplitudes at Fz, where MMN is expected to be largest, were only evident between the ignore and passive task and between the ignore and focused task, but not between the passive and focused task. The passive and focused task differed only over central and parietal regions. Thus, an N2b effect might have contributed to the differences between the ignore task, where no N2b is elicited, and the passive and focused task, where an N2b is probable. However, it appears implausible that the contribution of N2b is critical for the found differences between the tasks. Since N2b is highly dependent on attention, larger amplitudes would be expected in the focused task than in the passive task, which was not found in the present data. In addition, we found the same pattern of significant differences between the ignore task and the passive/focused tasks at the mastoid electrodes and this polarity reversal is typical for MMN only and does not occur for N2b, thus allowing for an estimation of the MMN effect independently from an N2b (Muller-Gass et al., 2005). As a result, we assume that MMN amplitudes did vary between the ignore task on the one hand and the passive and focused task on the other hand.

It has previously been suggested that two distinct components contribute to the elicitation of an MMN: one right-hemispheric frontal and one supratemporal component (Näätänen and Michie, 1979; Näätänen et al., 2007). This model assumes that amplitudes recorded at the mastoids represent an estimate of the supratemporal component of the MMN without an overlap of N2b. The data of the present study revealed significant variation of these mastoid amplitudes as a function of attentional focus. However, only the frontal component of the MMN has previously been linked to attentional modulation (Rinne, 2001). Thus, albeit the present study was not designed to test these models, the data indicate that also the supratemporal component is influenced by attention.

To sum up, the data render the MMN a potential tool to detect basic cognitive functions in the absence of directed attention. The MMN partially overlapped with an N1 in the ignore task and with an N2b in the passive and focused task. Despite this overlap, our data suggest that not only N2b but also the MMN was influenced by our attentional instructions. We assume that the process of standard formation (Sussman, 2007) was affected by the tasks. Of course, Sussman's model (2007) is only one of several possible MMN models, and in its details it remains controversial. However, it provides an explanatory framework for how attentional factors affect MMN amplitudes.

The three tasks required different levels of attention to be allocated to the auditory stimuli, resulting in different levels of subjective effort. Interestingly, our prediction of the highest subjective effort in the focused task, lower effort in the passive task, and the lowest effort in the ignore task was not confirmed. Subjective effort in the passive and focused tasks was equally high. Thus, listening to the stimuli without mental and behavioral engagement was judged to be as effortful as having to respond to each word pair or sentence, or monitoring the tone stream for odds. However, we assume that our passive task, especially when directly compared to the ignore and focused tasks, shared commonalities with vigilance and sustained attention tasks, which are characterized by a low rate of relevant stimuli and require concentrated attention over a prolonged period of time (e.g., Haga, 1984; Warm et al., 1996; Noyes, 2009). A vigilance decrement in such tasks (Colquhoun and Baddeley, 1964) leads to a drop in performance, sometimes already within 5 min after the initiation of the task (for a review, see Warm et al., 2008a). Warm and colleagues rejected the previous view that the decline of arousal and performance is exclusively due to monotony (Warm et al., 2008b). Instead, they concluded that vigilance tasks require a large amount of information processing, and are exhausting and capacity draining. Therefore, individuals experience high levels of subjective workload and stress (Grier et al., 2003). In support of these findings, Eastwood et al. (2012) defined the aversive state of boredom as correlating with high mental effort in terms of attentional processes: boredom arises when individuals are unable to engage attention to internal or external information, focus on the fact of this unsatisfactory state, and consider the reason for this state to be caused by the environment. Thus, participants in our study may have experienced boredom in the passive task.

However, there are alternative explanations for the ERP response decrement in the passive task. It is possible that psychological states such as frustration and mood led to an attenuation of the ERP components (Nijboer et al., 2010; Kübler et al., 2011). Furthermore, the instructions might have caused the participants to allocate different levels of motivation to the tasks, which can also influence ERP responses (Johnson, 1986; Kleih et al., 2010).

Taken together, in our study ERPs can be considered a measure of performance, which has been shown to decrease in vigilance and sustained attention tasks that are similar to our passive task. Although our passive task was shorter and simpler than the usual vigilance task, some characteristics are similar in both, such as a low level of signal input, and having to sustain attention over a prolonged period of time (up to 15 min in the sentence paradigm), especially when directly compared to the other, more engaging, ignore, and focused tasks. Furthermore, the high information processing requirements associated with those tasks led to high subjective effort in the passive task.

The presented results bear the limitation that no correction was performed for multiple comparisons of amplitudes between the tasks. We refrained from a correction procedure because the *t*-tests were pre-planned and tested directional assumptions defined in our hypotheses. In addition, correction methods may reduce the probability of Type I error, but often increase the probability of Type II error to an unacceptable level (Rothman, 1990; Nakagawa, 2004). To overcome this issue, we reported effect sizes as recommended by Nakagawa (2004).

Our results have important implications for applying such ERP-based paradigms in non-responsive patients for assessment of their cognitive functioning. Firstly, passive instructions in healthy participants led to high subjective effort, which may be caused by the passiveness being experienced as stressful and straining. In DoC patients whose attention span is much shorter than that of healthy individuals, required sustained attention in the absence of attention attractors may result in failed task performance and a consequent lack of the respective ERP components despite the intact neuronal sources. Secondly, instructions to passively listen to the stimuli attenuated ERP effects in the semantic paradigms while ERPs in the oddball paradigm were equally large in the passive and focused task. These weakened semantic ERPs could be attributed to the vigilance decrement due to the high mental effort and feelings of boredom caused by the monotonous situation. On the other hand, the reduction might have been caused by the lack of behavioral data in the passive task, thereby making it impossible to eliminate error trials with presumed low vigilance like we did in the focused task. An attenuation of semantic ERP effects is problematic if the passive instruction “just listen” is used in the assessment of non- or low-responsive patients. In this case, the expected differences in ERP components might be difficult to find even though the same stimuli would elicit large ERP effects under an active instruction specifically engaging the participant's attention in a goal-directed task. Particularly at the single subject level, where the signal/noise ratio is relatively low, the aim of stimulation paradigms has to be the elicitation of strong and robust ERP differences, which in semantic paradigms are unlikely to occur without active instructions. In our oddball paradigm, we found an attentional effect also on MMN amplitudes obtained in the ignore vs. passive and focused task. Even though further research is needed to clarify N1 and N2b overlaps, our results revealed smaller amplitudes in the traditional ignore task than in the passive or focused task.

## Conclusion

The presents study revealed attentional effects on N400 and MMN. N400 amplitudes gradually decreased with decreasing attention, leading to an extinction of the N400 in the ignore task. An MMN was present in all tasks but amplitudes were higher in the focused and passive tasks as compared to the ignore task. This is also supported by mastoid amplitudes indicating an attentional effect on the supratemporal component if the MMN.

In healthy participants, the passive instruction to “just listen” without providing a specific task regarding the stimuli leads to a high subjective effort and attenuated semantic ERP responses as compared to the instruction to fully concentrate and respond to auditory stimuli. In respect to the hierarchical approach, a passive instruction attenuated ERPs when applied to semantic material that required more processing recourses, as reflected by the N400. On the other hand, a passive instruction did not attenuate ERPs when applied to a simple tone stream that required only basic processing resources, as reflected by the MMN. In DoC patients, the exact level of awareness and thus the ability of the patient to follow instructions are often unknown. However, when assessing patients with ERP paradigms, we assume that the respective cognitive function is present and thus, an instruction to actively perform a task can be followed. In cases where the patient has no awareness, the instruction would not make a difference to the person. If awareness is present, however, an active instruction might help the patient to focus and consequently to exhibit larger ERPs, which will be easier to detect and which might contribute to conclusion about the cognitive state of the patient.

### Conflict of interest statement

The authors declare that the research was conducted in the absence of any commercial or financial relationships that could be construed as a potential conflict of interest.
